# Cryptic diversity: Two morphologically similar species of invasive apple snail in Peninsular Malaysia

**DOI:** 10.1371/journal.pone.0196582

**Published:** 2018-05-07

**Authors:** Suganiya Rama Rao, Thor-Seng Liew, Yoon-Yen Yow, Shyamala Ratnayeke

**Affiliations:** 1 Department of Biological Sciences, School of Science and Technology, Sunway University, Selangor, Malaysia; 2 Institute for Tropical Biology and Conservation, Universiti Malaysia Sabah, Kota Kinabalu, Sabah, Malaysia; University of Minnesota, UNITED STATES

## Abstract

Invasive snails in the genus *Pomacea* have spread across Southeast Asia including Peninsular Malaysia. Their effects on natural and agricultural wetlands are appreciable, but species-specific effects are less clear because of morphological similarity among the species. Our objective was to establish diagnostic characteristics of *Pomacea* species in Malaysia using genetic and morphological criteria. The mitochondrial COI gene of 52 adult snails from eight localities in Peninsular Malaysia was amplified, sequenced, and analysed to verify species and phylogenetic relationships. Shells were compared using geometric morphometric and covariance analyses. Two monophyletic taxa, *P*. *canaliculata* and *P*. *maculata*, occurred in our samples. The mean ratio of shell height: aperture height (*P* = 0.042) and shell height: shell width (*P* = 0.007) was smaller in *P*. *maculata*. *P*. *maculata* co-occurred with *P*. *canaliculata* in five localities, but samples from three localities contained only *P*. *canaliculata*. This study is the first to confirm the presence of two of the most invasive species of *Pomacea* in Peninsular Malaysia using a molecular technique. *P*. *canaliculata* appears to be the more widespread species. Despite statistical differences, both quantitative and qualitative morphological characteristics demonstrated much interspecific overlap and intraspecific variability; thus, shell morphology alone cannot reliably verify species identity. Molecular techniques for distinguishing between these two highly invasive *Pomacea* species are needed to understand their specific ecological niches and to develop effective protocols for their management.

## Introduction

Invasive species are one of the key threats to global biodiversity and are gradually altering aquatic and terrestrial communities worldwide [1, 2]. These species have imposed huge costs to fisheries, forestry, and agriculture by causing the decline of native species in natural environments [3, 4]. Among these invasive species, aquatic gastropods such as *Pomacea canaliculata* and *Pomacea maculata* (synonym: *Pomacea insularum*), sometimes referred to as golden apple snails collectively, are known to be especially difficult to manage and control [5–7].

*Pomacea canaliculata*, the channelled apple snail, and *P*. *maculata*, the island apple snail, are native to tropical South America and were introduced to other regions of the world, including tropical Asia through the food and aquarium trade [8–10]. *Pomacea*’s first introduction to Asia was presumably to Taiwan [9, 11]. Eventually, the snails were released into natural habitats and spread rapidly into paddy fields and natural wetlands in Asia where they destroyed crops and depleted wetland macrophytes, possibly altering ecosystem function [6, 8, 9, 12–17]. The Global Invasive Species Database lists *P*. *canaliculata* among the world’s 100 worst invaders [18]. *Pomacea* has attracted worldwide attention because they cause hundreds of millions of dollars of direct loss in rice agriculture and indirect loss in human health and ecosystem damage [4, 8, 19–22].

Previous studies suggested a number of diagnostic shell characters that can be used to differentiate *P*. *maculata* and *P*. *canaliculata* (Table 1). For example, “inner pallial lip pigmentation where *P*. *maculata* has red or orange pigmentation but not *P*. *canaliculata*, a thicker shell for *P*. *maculata* compared to *P*. *canaliculata*, and a higher spire for *P*. *canaliculata* compared to *P*. *maculata*” (Table 1) [17, 23]. However, biologists have reported difficulties in the use of shell characters for species identification because both species share many similarities and also display much intraspecific variability in shell morphology [23–26] Furthermore, *Pomacea* exhibits a high degree of geographic variation in many characters of shell morphology, such as shell color, pallial lip pigmentation and shell thickness [25, 27].

**Table 1:** Potential diagnostic shell characters to differentiate *Pomacea canaliculata* and *P*. *maculata*.

Qualitative characteristic	*P*. *canaliculata*	*P*. *maculata*	Reference
Pallial lip pigmentation (yellow/orange/red)	Unpigmented	Pigmented	[17, 23]
Shoulder	Rounded	Angulated	[17, 23]
Shell surface	Smooth	Rough	[23]
Suture	Deep	Shallow	[17]
Shell thickness (mm)*	Thin (< 0.905)	Thick (≥ 0.905)	[23]
Umbilical width	Wide	Narrow	[17, 23]
Shell length (mm)	35–60	35–165	[23]
Spire height: shell height (mm)	0.07–0.16	0.026–0.114	[23]

* The median for shell thickness measured 1 cm above the pallial margin (Fig 2) was 0.905 mm.

Measurements < 0.905 mm were classified as thin and ≥ 0.905 mm were classified as thick.

To aid the identification of *Pomacea* species, molecular tools based on a portion of the mitochondrial gene, cytochrome oxidase subunit I (COI), have been developed and tested in Japan, Australia and Thailand [25, 26, 28, 29]. One approach is to sequence the DNA of the standard COI region and the percentage similarity of genetic sequences are compared to sequences uploaded on GenBank. A second approach is a rapid molecular method where specific primers are used to amplify particular regions in the COI gene and the length of the PCR fragments are compared on a gel image. Both molecular tools help to differentiate among a few common and morphologically similar species of *Pomacea*, which allows subsequent investigation of species distributions and morphological variation within and between species.

In Malaysia, studies on *Pomacea* have focused on their distribution, impact, and control in rice fields [17, 30–35], but no comprehensive studies have been conducted on their general distribution, impacts to natural wetlands, and taxonomy. Accurate identification of species is an essential first step towards evaluating their individual ecological and economic impacts and developing appropriate control and eradication measures [28, 36]. Many reports of the impacts caused by species of *Pomacea* may be confounded by misidentification because species identification was based on conchology [6]. Hayes et al. [23] reported qualitative and discrete conchological (including internal anatomical) features to distinguish the two species in their native environment and a small part of their introduced range in the southern United States. Nevertheless, *Pomacea* demonstrates much phenotypic plasticity, suggesting that conchological characteristics may differ in new invaded environments [37]. Thus, shell characteristics that sufficiently distinguish species in some geographic locations, may be less effective in others.

In view of the paucity of knowledge on *Pomacea* species in Malaysia, we used available molecular tools to: (1) confirm the two species, namely *P*. *canaliculata* and *P*. *maculata*, which occur sympatrically in some areas by using a rapid PCR protocol; (2) estimate the molecular phylogeny of populations of *Pomacea* species in the context of *Pomacea* species from other regions based on the mitochondrial COI gene; and (3) investigate shell morphological variation of both species in term of size, shape and qualitative shell characters. Our results suggest that *P*. *canaliculata* and *P*. *maculata* occur in Malaysia and both species cannot be identified solely based on diagnostic conchological characters as suggested by previous studies. The high degree of morphological variation within species and interspecific overlap, especially in sympatric populations, may hinder species identification, which should ideally precede subsequent research on *Pomacea* in Malaysia.

## Methods and materials

### Field collection and sample processing

**Fig 1:**
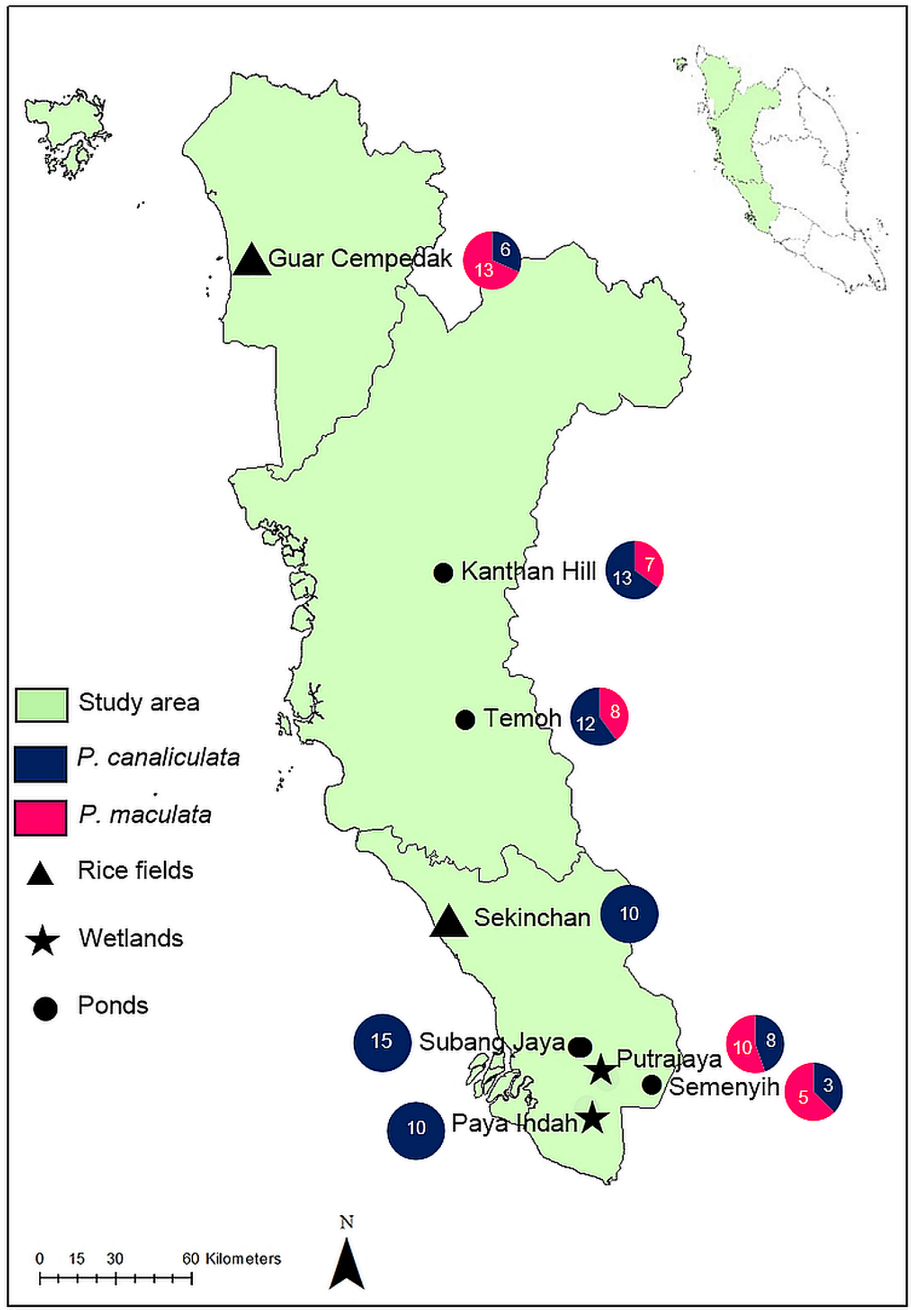
Geographic locations in Peninsular Malaysia where *Pomacea* specimens were collected February 2016—November 2016. Three to five sites at each location were surveyed to collect 10 to 15 of the largest snails. *P*. *canaliculata* occurred at all sites, including Semenyih, Kanthan Hill, and Guar Cempedak, but not *P*. *maculata*. Pie charts display prevalence of each species in the sample after genetic screening. Symbols represent habitats.

*Pomacea* specimens were collected from freshwater wetlands such as ponds, lakes and rice fields, at eight geographical locations in Peninsular Malaysia from February to November 2016 (Fig 1 and S1 Table). At each geographic location, we collected 10–15 of the largest available *Pomacea* specimens from 3–5 spatially discrete sites. The Malaysian environment is aseasonal with warm, humid conditions; thus snail survival was assumed to be similar at different times of the year. No specific permission was required for these locations and activities because the study was not conducted in protected areas and did not involve endangered or protected species. Species identity based on morphology was not attempted at this stage. Snails were dispatched using heat shock [23], which facilitated extraction of the entire soft body leaving the shell intact. Approximately 1 g of foot tissue was removed and preserved in ~ 500 μl of absolute ethanol for DNA extraction. Shells were cleaned and labelled for morphological analysis.

### Genomic deoxyribonucleic acid (DNA) extraction

Approximately 1 to 5 mg of foot tissue was extracted from *Pomacea* specimens and immersed in distilled water for 2 hours to soften the fibres before DNA extraction. We included 38 specimens from a previous study [38]. Genomic DNA was extracted using the NucleoSpin® Tissue kit (Macherey-Nagel, Germany) according to the manufacturer’s instructions. The eluted DNA was stored at -20°C for future use. The yield and purity of isolated DNA were measured using Biodrop μlite (Biodrop, United Kingdom).

### A rapid method for polymerase chain reaction (PCR) amplification

The DNA extract of each specimen was subjected to two separate PCR protocols. The first was a rapid diagnostic protocol for species identification [25, 26, 39], in which multiplex PCR was performed to amplify a fragment of the mitochondrial cytochrome *c* oxidase subunit (COI) gene using two forward primers: PcanCOI (F) (5’-TGG GGT ATG ATC AGG CC-3’) and PinsCOI (F) (5’-ATC TGC TGC TGT TGA AAG-3’) and a reverse primer: HCO 2198 (R) (5’-TAA ACT TCA GGG TGA CCA AAA AAT CA-3’). A final volume of 25 μl was prepared for the amplification of DNA: 12.5 μl of Prime Taq Premix (2X) (GENETBIO Inc, Korea), 1 μl of 10 pmol forward and reverse primers, 1 μl of template DNA, and high quality distilled water to make up the total. We used the GeneAmp® PCR system 2700 (Applied Biosystem) thermal cycle with an initial denaturation at 94°C for 5 min followed by 36 cycles of amplification (denaturing 94°C for 30s, annealing at 55°C for 30s, extension at 72°C for 1 min) and lastly final extension at 72°C for 5 min [39]. PCR products were viewed on a 1% agarose gel. Larger PCR fragments (666 bp) represented *P*. *canaliculata* whereas smaller PCR product size (390 bp) represented *P*. *maculata*. Based on the results of this screening, we chose all available individuals (N = 26) of the rarer species (*P*. *maculata*) in our sample and 26 individuals of *P*. *canaliculata* for subsequent DNA sequencing and morphological analyses.

A second PCR was performed for each sample using the mitochondrial primers of the cytochrome c oxidase subunit (COI) gene LCO 1490 (F) (5’- GGT CAA CAA ATC ATA AAG ATA TTG-3’), HCO 2198 (R), [39] and the previously described thermocycler protocol. The PCR products were sent for sequencing at MyTACG Biosciences Enterprise, where samples were sequenced using BigDye® Terminator v1.1, v3.0 and v3.1 Sequencing Kit and analysed with Applied Biosystems 3730xl DNA Analyser.

To explore phylogenetic relationships of *Pomacea* species in Malaysia with *Pomacea* species in other geographic regions, we obtained several COI sequences of nine *Pomacea* species from the NCBI GenBank. Closely related sequences clustered into discrete clades, thus 2–3 representative sequences were selected from different countries from each clade to produce a final tree including 20 representative sequences (S1 Fig) Sequences were checked using Chromas 1.42 (Technelysium Pty.Ltd., Australia) and BioEdit 7.0.9.0 [40] software and aligned using CLUSTAL X program [41]. Akaike’s Information Criterion (AIC) was used to estimate the best fit model for each of the three codon positions for COI using Program jModeltest 2.1.6. The best fits were the TrN+I for 1^st^ codon, HKY+I for 2^nd^ codon, and TIM1+G for 3^rd^ codon. These parameters were used to specify the models of sequence evolution, and as priors for Bayesian analysis. Bayesian inference were run in MrBayes v.3.2.1 [42] on CIPRES portal [43] (Miller et al., 2010) with the following setting: mcmc negen = 10000000; nchains = 4; samplefreq = 100; average deviation of split frequencies < 0.01; and a burn-in value of 25%. The resulted phylogenetic tree was rooted by *P*. *scalaris* and *P*. *diffusa*.

### Morphological measurements and analysis

**Fig 2:**
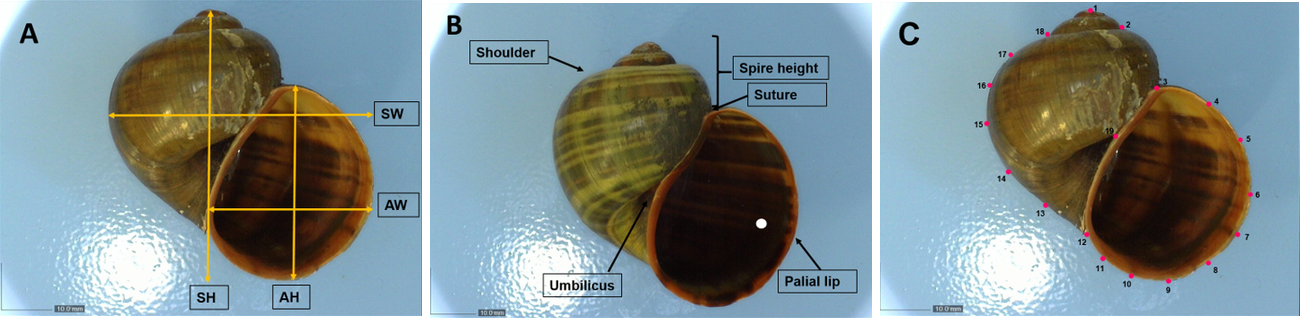
Shell characters used for morphological analysis. **(A)** Quantitative measurements obtained from aperture view of the shell: shell width (SW), aperture width (AW), aperture height (AH) and shell height (SH). **(B)** Shell qualitative characters (See Table 1 for details). **(C)** The location of 19 landmarks used in geometric morphometric analysis of shell shape.

We measured several quantitative and qualitative shell characters, including those previously suggested as diagnostic characters for *P*. *canaliculata* and *P*. *maculata* [23, 44]. Importantly, we wished to identify easily measurable characteristics to be used in the field. Five quantitative measurements of shell dimension, namely shell height, shell width, aperture height, spire height, and aperture width, were taken from photographs of a standardized aperture view of the shell using DinoLite imaging software (Fig 2, S2 and S3 Figs). Shell thickness, and umbilical width, were measured directly from specimens using digital callipers. In addition, five qualitative shell characters—pallial lip pigmentation, shoulder shape, shell surface texture, and depth of suture—were recorded (Fig 2, Table 1).

Variation in shell measurements is strongly influenced by differences in age, environment and consequently, size of individual snails. We checked data for normality using the Shapiro-Wilk test [45] before exploring quantitative shell measurements using principal component analysis [46] to identify those shell metric(s) that explained most of the variation in the data. We then examined interspecific differences using covariance analyses (ANCOVA; Sokal and Rohlf 1995) to control for differences in shell size. We used built in functions in Program R v. 3.1.3; [47] for ANCOVAs.

To compare shell shape between the two species, we used geometric morphometrics [48]. We identified 19 homologous landmarks from the specimen images (Fig 2) and obtained the coordinates of each landmark using ImageJ software [49] Version 1.05a. We used generalized procrustes analysis (GPA) to normalize the size of the shells, after which coordinates were analysed using principal component analysis. Package “geomorph” [50] in Program R v. 3.1.3;(46) (R Development Core Team 2014) was used to run the geometric morphometric analysis.

## Results

**Fig 3:**
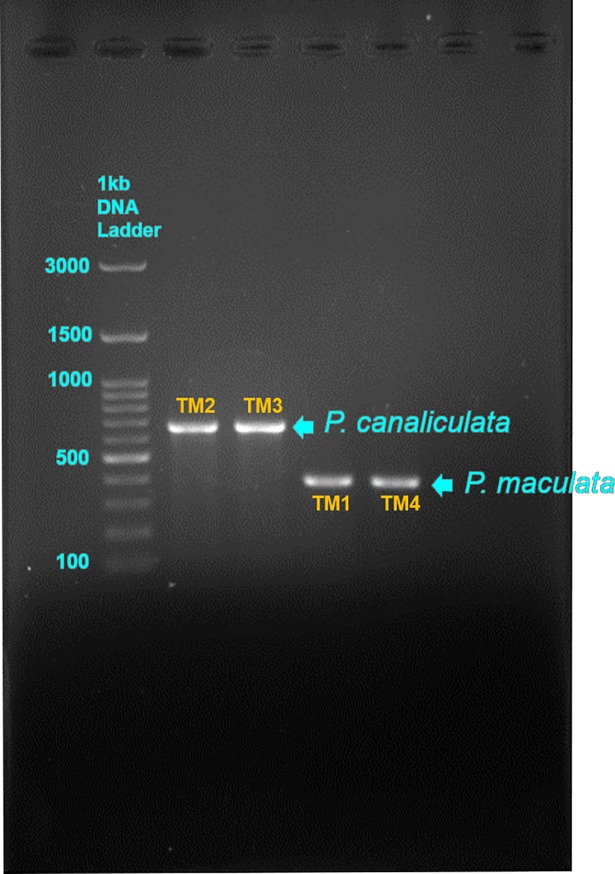
Gel electrophoresis image which shows length differences of the amplified genomic DNA fragments of *P*. *canaliculata* and *P*. *maculata* using the mitochondrial primers of the cytochrome c oxidase subunit (COI) gene PcanCOI (F) and PinsCOI (F), HCO 2198 (R). Amplified fragments were 666 bp and 390 bp respectively.

We identified two species of *Pomacea* in Peninsular Malaysia using the rapid PCR diagnostic method (Fig 3). Gel electrophoresis of PCR products generated two categories of amplicon lengths: a larger fragment of 650–700 bp, and a smaller fragment slightly < 400 bps. The two bands corresponded with the species identified by [25]: 666 bp for *P*. *canaliculata* and 390 bp for *P*. *maculata*. Based on this screening, 26 individuals of each species were selected for further analysis. *P*. *canaliculata* was the more widespread and abundant species occurring at all eight geographic locations, whereas *P*. *maculata* occurred at five locations, but always sympatric with *P*. *canaliculata*. Thus, at Guar Cempedak, Semenyih and Kanthan Hill, only *P*. *maculata* specimens were selected to achieve symmetrical sample sizes for both species (Fig 1).

### Molecular phylogeny of *Pomacea* species in Peninsular Malaysia

**Fig 4:**
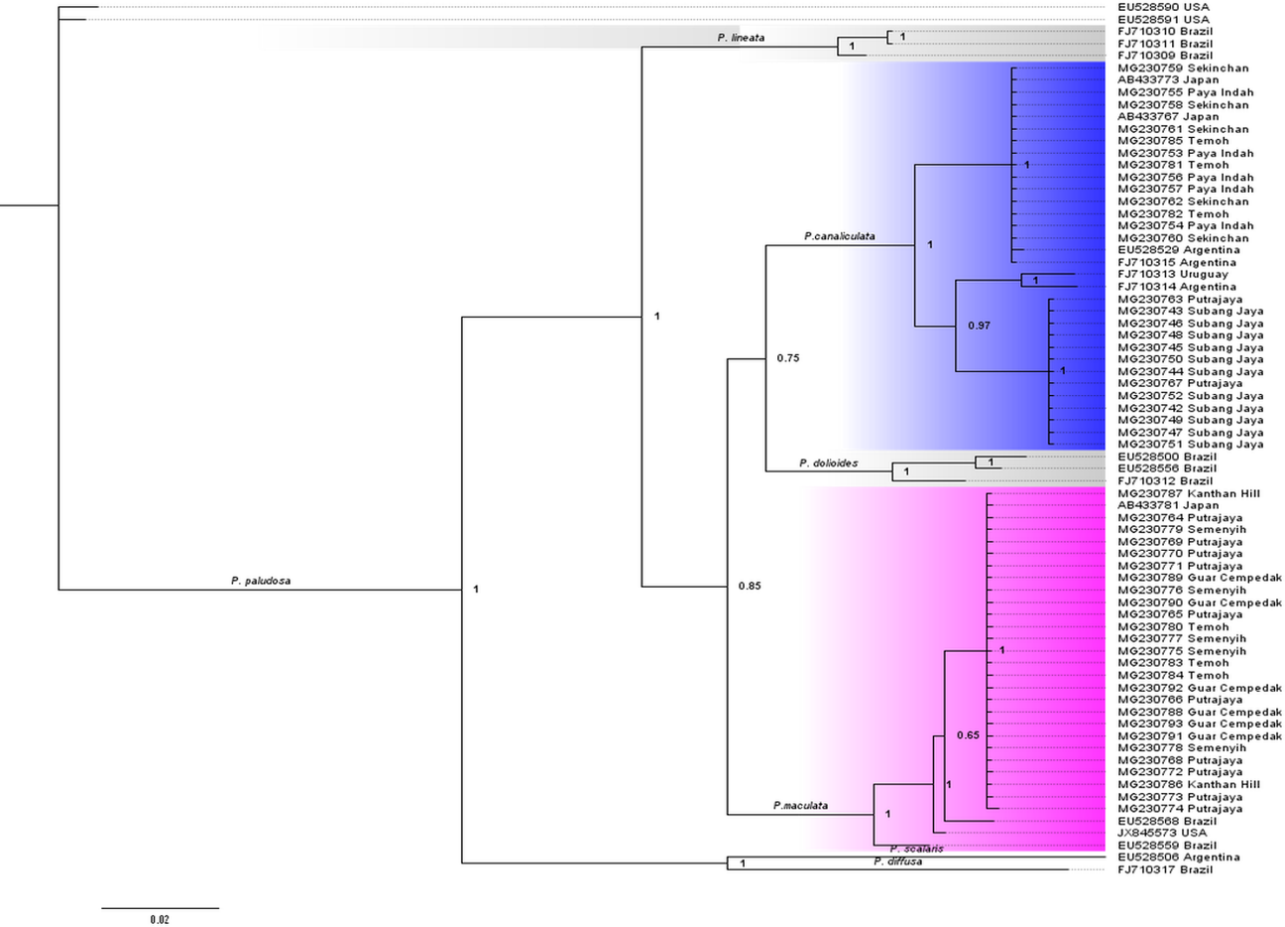
Phylogram shows BI analysis of *Pomacea canaliculata* and *P*. *maculata* in Peninsular Malaysia with other *Pomacea* species from introduced (USA, Japan) and native (South America) ranges. Bayesian posterior probabilities are shown for each clade. *Pomacea scalaris* and *Pomacea diffusa* were selected to root the tree. Only nodes with Bayesian posterior probabilities (PP) greater than 0.5 as a validity supported clade for the construction of a 50% majority rule consensus tree.

The length of the COI gene for *Pomacea* from selected localities in this study varied from 690 to 718 bp. The downloaded sequences of COI gene from GenBank varied from 650 bp to 690 bp. The lengths of all sequences were standardized by complete alignment for the phylogenetic analysis and the aligned sequences were used to generate a Bayesian Inference tree consisting of 594 characters and 72 taxa (Fig 4). All *Pomacea* snails from Peninsular Malaysia clustered into two monophyletic clades. Clade 1 clustered with *P*. *canaliculata* from Japan, Argentina and Uruguay. Clade 2 shared the same haplotype with *P*. *maculata* from Japan, Brazil and USA. The phylogenetic analysis based on the COI sequences confirmed the results of the rapid PCR diagnostic method; two species, *P*. *canaliculata* and *P*. *maculata*, comprised our sample.

### Shell morphology

**Fig 5:**
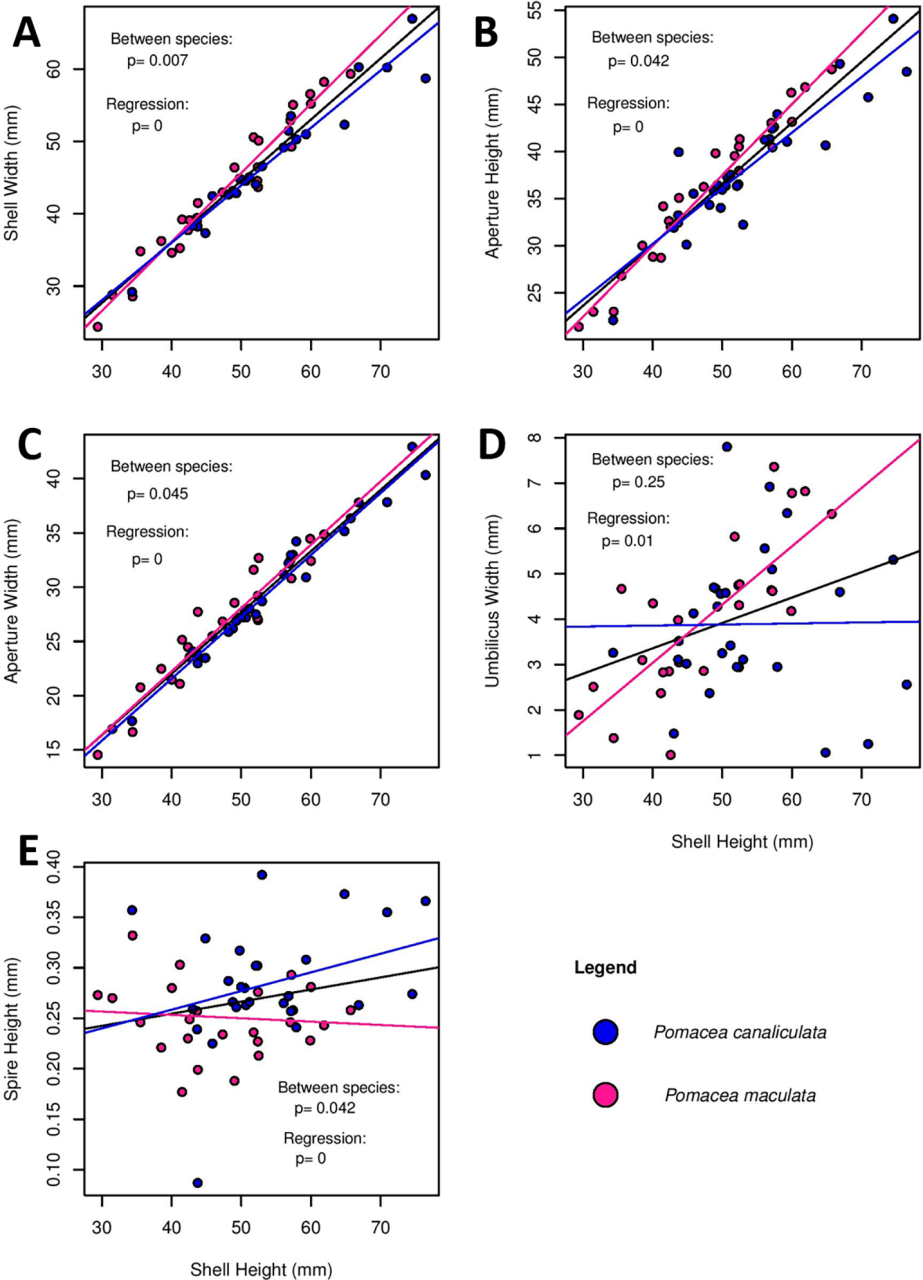
Regressions of different discrete shell characteristics of *Pomacea canaliculata* (N = 26) and *P*. *maculata* (N = 26) as a function of shell height. Statistical probabilities are shown for the effect of shell height on the dependent variables (regression), and to test the hypothesis that the two species differ. The black regression line represents the combined data of both species. Regression slopes for all metrics were positive and significant, thus shell height had a strong influence on other shell metrics. Species differences are evident in all except umbilical width.

Normality tests for all raw data of shell measurements were non-significant (Shapiro test, p > 0.05). Principal component analysis of shell metrics indicated that 96.5% of the variation was explained in the first principal axis. In addition, the highest component loading for the first principal axis was shell height, which suggested it could be used as a proxy for overall shell size (S2 Table). Thus, we used shell height as a covariate in regression analyses of all remaining linear metrics of shell size. All five shell measurements showed significant relationships with shell height (Fig 5). When species effects were examined, shell width showed the most marked statistical difference (Fig 5A), with *P*. *maculata* tending to have a slightly wider, or rounder shell. There were no statistical significant differences in umbilical width between the two species. However, there were marginal species differences (p = 0.042–0.045) of other shell measurements (Fig 5B, 5C and 5E). For instance, shells of *P*. *canaliculata* tending to have a higher spire, and a shorter, narrower aperture than *P*. *maculata* when variation in shell height was accounted for. Despite statistical differences, the slopes of the species regression lines intersected for all except Fig 5C, and data points for both species overlapped, even after standardizing for shell height. Thus, linear shell measurements may have little practical value as diagnostic characters. Full results of the ANCOVA are in S1 Table.

#### Qualitative shell characters

**Fig 6:**
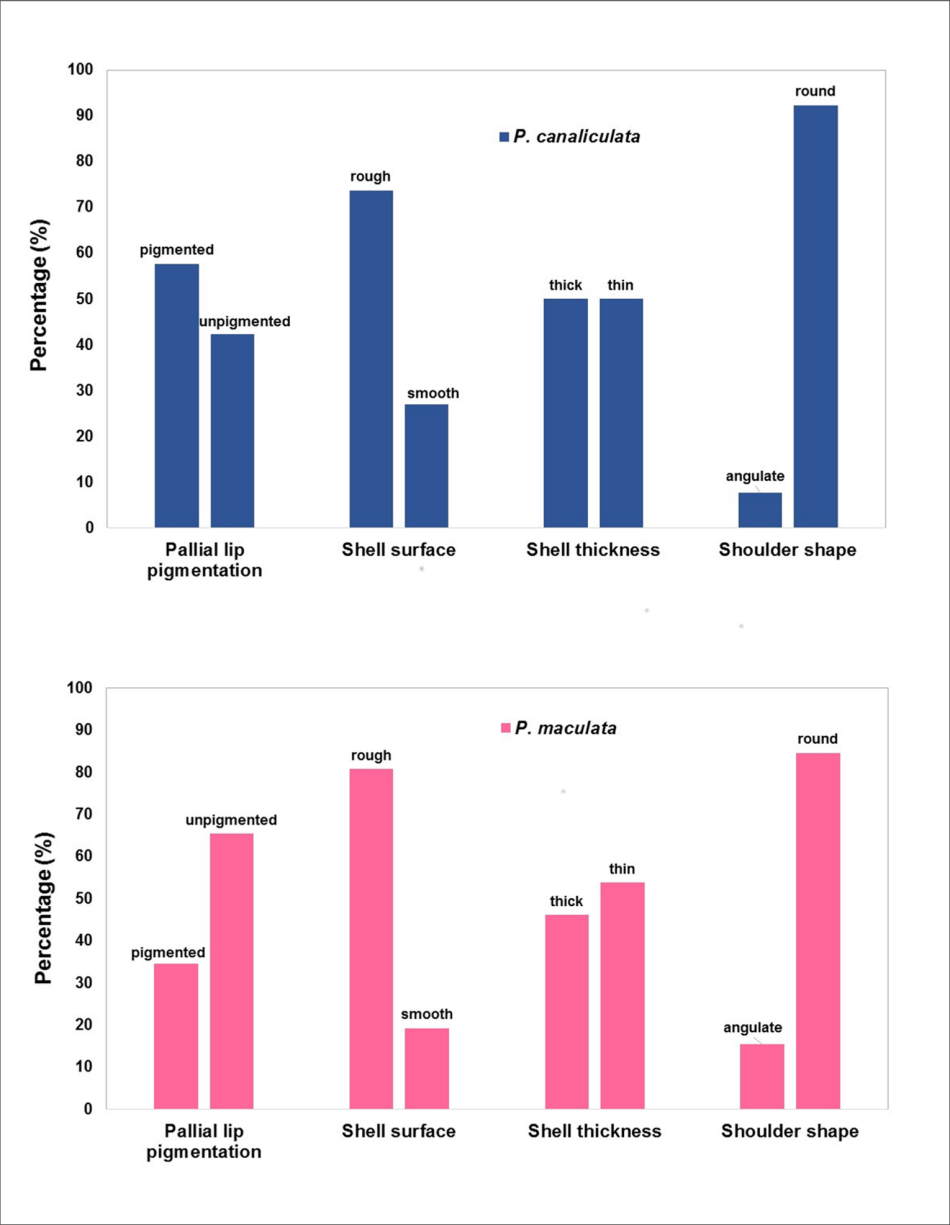
Comparison of qualitative characteristics between 26 *Pomacea canaliculata* and 26 *P*. *maculata* shells collected in Peninsular Malaysia.

None of the qualitative characters we examined in our sample in this study were unique to a species (Table 1, Fig 6). Suture depth was not displayed because all snails in the sample had deeply channelled sutures.

#### Geometric morphometric analysis

**Fig 7:**
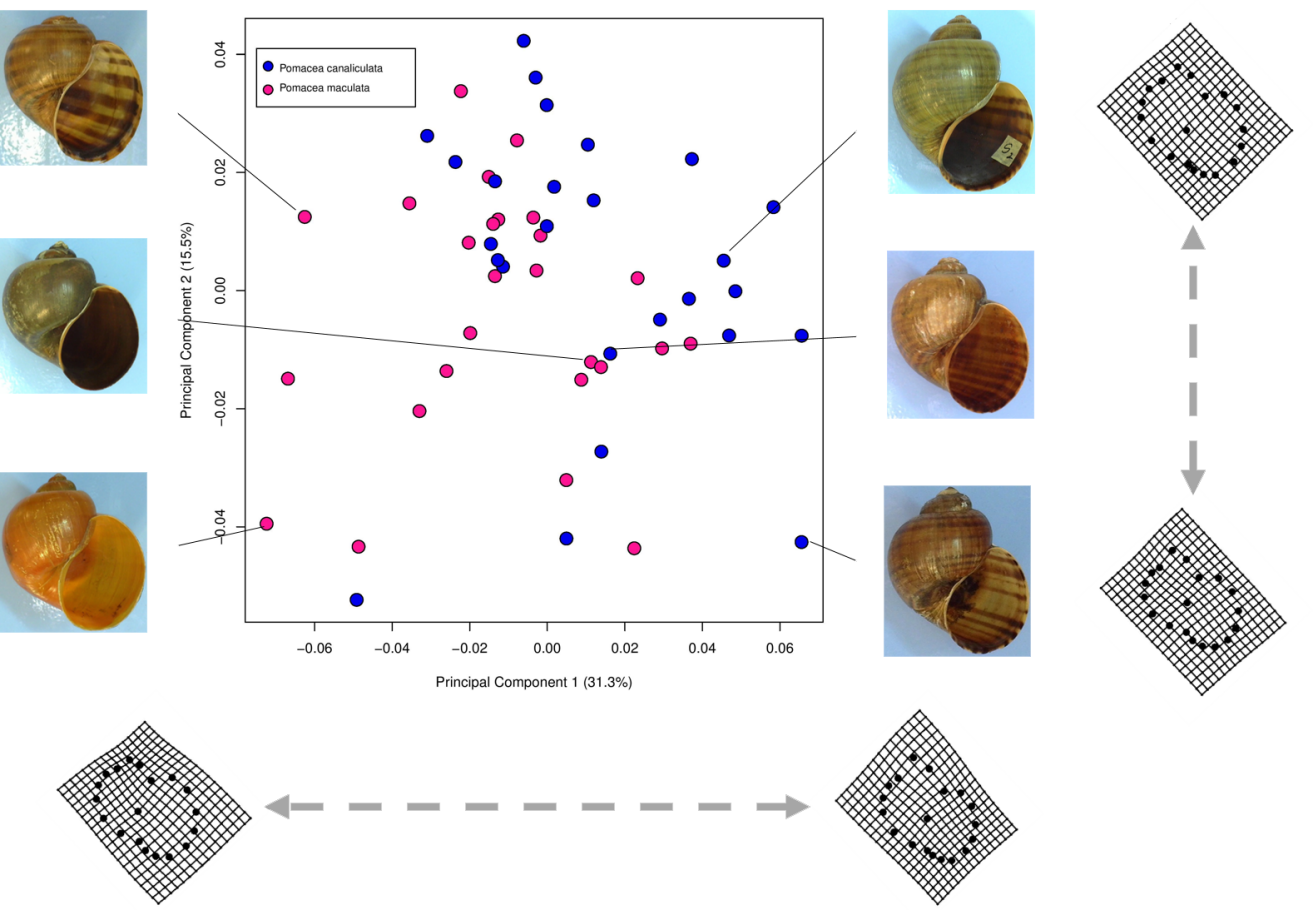
Morphospace represented by scatterplot of the scores of the first two principal components of the geometric morphometric analysis of the shell’s 19 landmarks. Representative shells in the morphospace are shown. Thin-plate spline (TPS) transformation grids represent shape variation at the extremes of the principal component axes.

The first two principal components of geometric morphometric analysis explained a total of 46.8% (PC 1–31.3%; PC 2–15.5%) of the shape variation of the 19 shell landmarks dataset. Shell shape variation along axis principal component 1 mirrored a more pointed shell (i.e. with a relatively higher spire) and a smaller aperture relative to the shell whorl at the positive end of the axis, and a less pointed (i.e. relatively lower shell spire) and larger aperture in relative to the shell whorl at the negative end of axis. The two *Pomacea* species show much overlap in the scatterplots (Fig 7). Therefore, no clear differences in shell shape were evident.

## Discussion

In this study, we expected that two species of *Pomacea*, namely, *P*. *canaliculata* and *P*. *maculata* occurred in Malaysia as previously recorded in rice fields in Peninsular Malaysia [17, 33,35]. Studies in other parts of the world have established that several species in genus *Pomacea* are clearly delimited by phylogenetic analysis [25, 28, 51]. This study is the first to use molecular genetics to confirm species identity in Malaysia by using a rapid diagnostic method [26]. Phylogenetic analysis reconfirmed the presence of *P*. *canaliculata* and *P*. *maculata* in the geographic areas that we surveyed (northern and central of Peninsular Malaysia).

Although our inference is based on a limited sample size, the presence of two haplotypes in *P*. *canaliculata*, and only one haplotype for *P*. *maculata* suggest two possible introduction events. Furthermore, *P*. *canaliculata* occurred in all eight locations surveyed in this study and was sympatric with *P*. *maculata* in five of those locations. Based on the wider distribution and catch frequencies (including data in preparation), *P*. *canaliculata* is more abundant and widespread in Peninsular Malaysia than *P*. *maculata*, a possible consequence of being introduced earlier to the Peninsular and having a longer period to establish and spread.

Despite the genetic analysis clearly demarcated the species of *Pomacea* in our study, species identification based on shell characteristics was difficult. Analyses of traditional quantitative shell characteristics such as shell width, or aperture height, using shell height as a covariate, demonstrated interspecific differences; *P*. *maculata* shells possessed a slightly greater ratio of aperture height to overall shell height, thus a more rounded shell with a shorter spire relative to *P*. *canaliculata*. However, the magnitude of the differences was small and both species overlapped to the extent that they possessed little practical value to reliably separate *P*. *maculata* from *P*. *canaliculata*. Even, umbilical width, which has been used as a qualitative species diagnostic character [17], varied greatly within and between species of snails in our study.

Furthermore, several widely used qualitative characteristics that distinguish *P*. *canaliculata* from *P*. *maculata* [17, 52] failed to do so in our study. For example, the red/pink/orange pigmentation of the inner pallial lip, which is characteristic of *P*. *maculata* in its native range [23] was observed more frequently in shells of *P*. *canaliculata* in our sample. In our study, thin and thick shells, including smooth- and rough-textured shells, occurred in both species in similar proportions, almost all shells possessed a rounded shoulder, and all shells possessed deeply channelled sutures with no noticeable difference between species. Shell thickness and surface characteristics in *Pomacea* and other ampullariids is reportedly variable within species, and may be associated with calcium content and aquatic pH [24, 37, 53]. Although we did not analyse morphometric data by geographic location, we noticed variation in shell thickness by locality and speculate that environmental variation including age of the snail likely obscured species differences.

Many studies have demonstrated that where traditional morphometrics using linear measurements fail to decipher morphological differences, geometric morphometric approaches that capture the spatial configuration of landmarks on specimens have proved to be much more informative [48], especially for discriminating among cryptic species (e.g Mitrovski et al., [54]). In our study, geometric morphometric analyses confirmed broad overlap between *P*. *maculata* and *P*. *canaliculata* in shell shape, in accordance with the results of the linear measurements. Thus, neither qualitative nor quantitative shell characteristics were helpful for discriminating *P*. *maculata* from *P*. *canaliculata* in our sample. It is noteworthy that in their native range, *P*. *canaliculata* and *P*. *maculata* demonstrate qualitative and discrete conchological characteristics sufficient to separate them as species [23], but fail to do so in the Malaysia environment. One possibility is that different selection pressures in the Malaysian environment have resulted in convergence toward a particular phenotype and behaviour; future studies among different habitat types and environmental gradients will be needed to test this hypothesis. Regardless of the reasons, our results underscore the need for systematic records and species descriptions of *Pomacea* by geographic region to improve the prospects of reliable species identification.

An important consideration is that the two haplotypes corresponding to *Pomacea canaliculata* and *P*. *macula*ta in Malaysia may be hybrids rather than mixtures of two distinct species. *P*. *maculata* COI haplotypes were always found with *P*. *canaliculata* COI haplotypes in our samples, whereas populations consisting purely of *P*. *canaliculata* haplotypes also occurred. Yang et al. [55] found a similar species distribution pattern for *P*. *canaliculata* and *P*. *maculata* in China, although they do not report data on conchological characteristics and morphometric overlap. Matsukara et al. [25] commented on two qualitative shell characteristics, shell colour and spiral banding, with subtle differences in the intensity of spiral bands in the shells of *P*. *canaliculata* and *P*. *maculata* collected from the same location in Japan. Subsequently, Matsukara et al. [56] reported possible genetic exchange between the species based on sequence analysis of the nuclear gene elongation factor 1-alpha (EF1α) from snails collected from a variety of Southeast Asian locations, including their native range in Argentina. Mating experiments in the lab confirmed that the two species were capable of hybridizing with viable F1 progeny, but showed reduced hatchling success compared to matings among pure strains [56]. Notably, their results suggested that genetic exchange may have occurred, and may be ongoing, in both native and invaded populations. Comparisons of shell characteristics were not reported in this study, but genetic exchange was inferred in <20% of the snails, and egg masses in the field clearly differed between species; Matsukara et al. [56] concluded, therefore, that *P*. *canaliculata* and *P*. *maculata*, for the most part, maintained species-specific populations in East and Southeast Asia. Nevertheless, their results suggest that the potential for, and extent of, hybridization may explain why discriminating between phenotypes of *P*. *canaliculata* and *P*. *maculata* is sometimes possible (e.g., Hayes et al. [23]), and sometimes not, as in Peninsular Malaysia.

Hybridization has important implications for invasion success if traits acquired from parents confer greater fitness and consequently greater invasive success in offspring [57]. Potential consequences include novel life-history traits [58], or the ability to invade new territory and tolerate conditions that the parent cannot [59] often resulting in the extinction of one or both of the parent species [60]. Putative hybrids of *Pomacea canaliculata* and *P*. *maculata* in their native range demonstrate intermediate tolerance to two environmental stresses, cold and desiccation tolerance, suggesting that hybrids will have greater invasive success than pure strains of *P*. *maculata*, but not *P*. *canaliculata* [61]. The potential for improved environmental tolerance and altered life history traits among *Pomacea* hybrids in the non-native range has not been reported to our knowledge. Assays of nuclear loci will help to verify whether hybridization occurs in sympatric populations of *Pomace*a in Malaysia. If so, understanding the ecology and life-history of hybrid populations in Malaysian habitats will be crucial for controlling *Pomacea’s* damage and spread.

Hayes et al. [23] noted that males of *P*. *maculata* and *P*. *canaliculata* differed internally in reproductive anatomy, namely differences in the number and location of glandular tissues in the apical penial sheath. Thus, morphologically, males of the two species may be distinguishable by dissection. Moreover, egg size, number of eggs per clutch, hatchling shell morphology and hatchling size reportedly differ, with *P*. *canaliculata* eggs and hatchlings approximately twice the size of *P*. *maculata* [23]. These characteristics may potentially be more useful than adult shell anatomy to differentiate between the two species, but will need to be verified regionally.

We did not separate snails based on sex and we did not separate analyses by geographic location. Our results conflict with reports of *P*. *maculata* being the more abundant and widespread species of *Pomacea* in rice fields of Tanjung Karang, Selangor [17, 35]. We found only *P*. *canaliculata* from samples (N = 48) from this area over 4 sampling periods, 2016–2017 (this study including unpublished data). Whether management protocols for snails in rice fields selectively reduced populations of *P*. *maculata*, or whether species were misidentified, is unknown. If the former, it underscores the need for reliable species identification, especially in regions where the snails have significant economic impacts.

The misidentification of species that impose economic or health costs can have severe consequences for pest management and human safety [62, 63] and hampers the ability to develop effective solutions to manage their populations [36]. However, the discovery of cryptic species, including cryptic species complexes, has increased rapidly since the availability of DNA sequencing to detect and differentiate morphologically similar species. *Pomacea maculata* and *P*. *canaliculata* display a high degree of morphological similarity in Peninsular Malaysia, but have clearly different mitochondrial haplotypes. Both species were identified from molecular sequencing data but were indistinguishable in shell characteristics, despite our concerted effort to use available descriptions, including geometric morphometric methods for species identification. Although we report data for 52 specimens in this study, wide-scale surveys in the peninsular, including repeated sampling of known sites (data in preparation), have provided consistent results of the presence of these two species and no additional species of *Pomacea* to date. Local environmental conditions, including historic or ongoing hybridization, may account for the phenotypic similarity of *P*. *canaliculata* and *P*. *maculata* in Malaysia. We suggest that the screening process [26] used in this study may be useful to obtain rapid verification of species identity, which should ideally precede studies of their ecology and management, including that of possible hybrid populations.

## Supporting information

S1 Table Summary detail of all the collected specimens. Sample collection coordinates, qualitative characteristics, shell measurements and landmarks coordinates for each specimen.
(CSV)

S2 Table Principal component analysis of shell metrics. (DOCX)

S1 Fig Bayesian inference of phylogeny. Numbers at nodes represents the Bayesian posterior probabilities.
(TIF)

S2 Fig Apertural views of each *Pomacea maculata* specimens in this study. (PDF)

S3 Fig Apertural views of each *Pomacea canaliculata* specimens in this study. (PDF)
