# Supplementary material for: Cryptic diversity: Two morphologically similar species of invasive apple snail in Peninsular Malaysia
Source: PLoS One. 2018 May 7;13(5):e0196582. doi: 10.1371/journal.pone.0196582 (PMC5937749; doi:10.1371/journal.pone.0196582)
Supplement: S2 Table — (DOCX) [file pone.0196582.s002.docx]

**Table S2. Principal component analysis of shell metrics.**

|  | PC1 | PC2 | PC3 | PC4 | PC5 |
| --- | --- | --- | --- | --- | --- |
| Standard deviation | 16.479 | 2.3485 | 1.52456 | 1.23750 | 0.6923 |
| Proportion of variance | 0.965 | 0.0196 | 0.00826 | 0.00544 | 0.0017 |
| Cumulative proportion | 0.965 | 0.9846 | 0.99285 | 0.99830 | 1.0000 |

|  | PC1 | PC2 |
| --- | --- | --- |
| Diameter of umbilicus (mm) | 0.04215515 | 0.39188826 |
| Aperture height (mm) | 0.42483645 | 0.56463108 |
| Shell height (mm) | 0.62241601 | 0.66936062 |
| Aperture width (mm) | 0.36267423 | 0.01743785 |
| Shell width (mm) | 0.54662844 | 0.28154505 |
