# Supplementary figures and images for: Cryptic diversity: Two morphologically similar species of invasive apple snail in Peninsular Malaysia
Source: PLoS One. 2018 May 7;13(5):e0196582. doi: 10.1371/journal.pone.0196582 (PMC5937749; doi:10.1371/journal.pone.0196582)

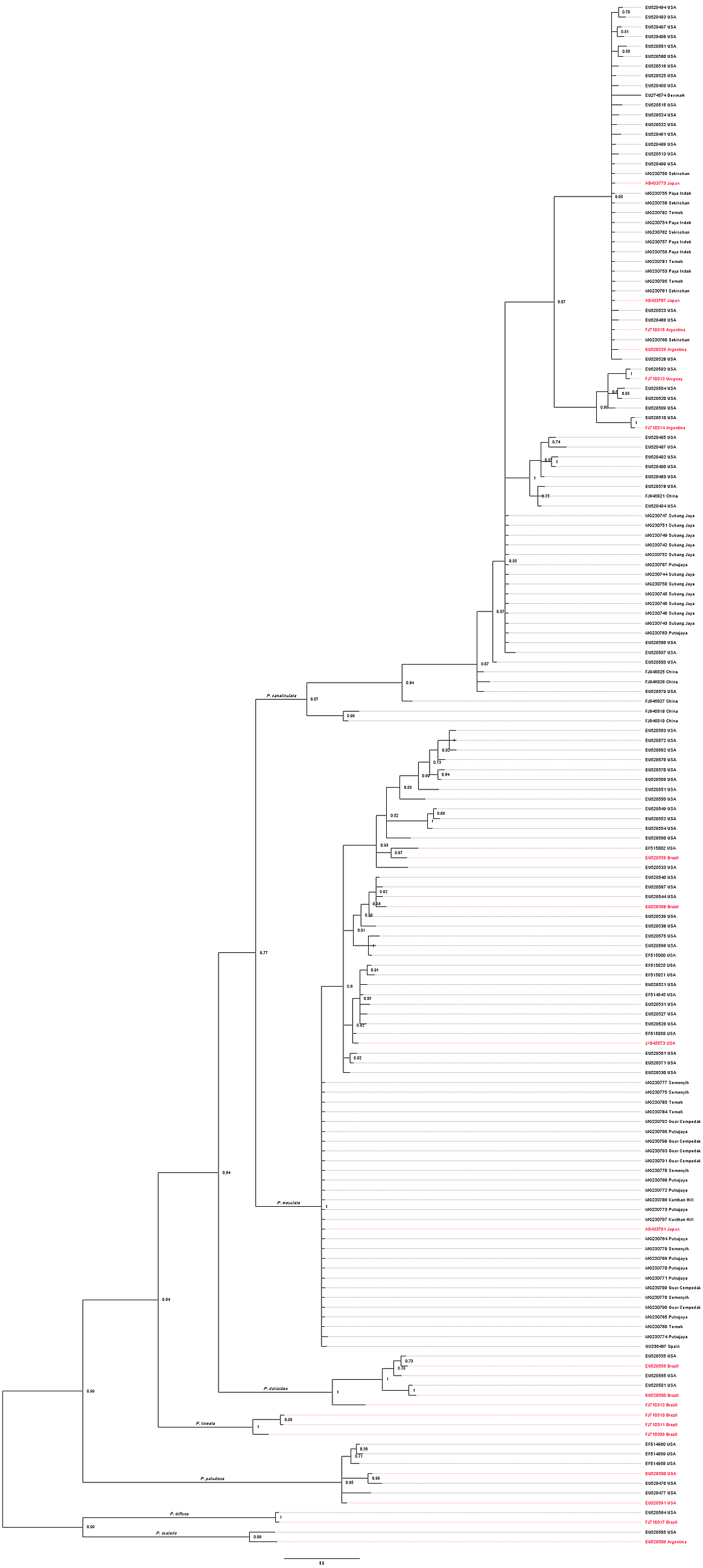

Supplement: S1 Fig — Numbers at nodes represents the Bayesian posterior probabilities. (TIF) [file pone.0196582.s003.tif]
