# Supplementary material for: Cryptic diversity: Two morphologically similar species of invasive apple snail in Peninsular Malaysia
Source: PLoS One. 2018 May 7;13(5):e0196582. doi: 10.1371/journal.pone.0196582 (PMC5937749; doi:10.1371/journal.pone.0196582)
Supplement: S2 Fig — (PDF) [file pone.0196582.s004.pdf]

S2 Fig. Apertural views of each *Pomacea maculata* specimens.

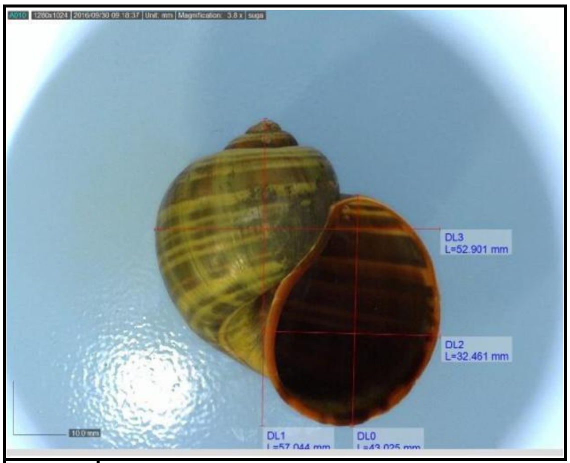

PJ 2

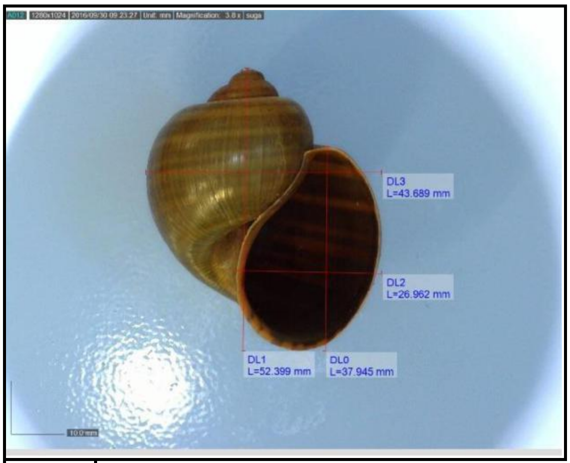

PJ 3

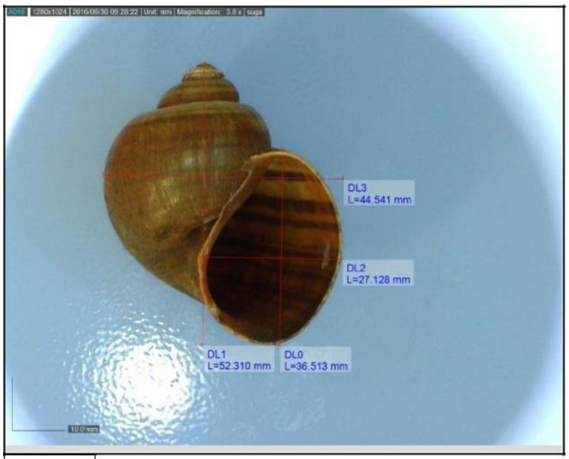

PJ 4

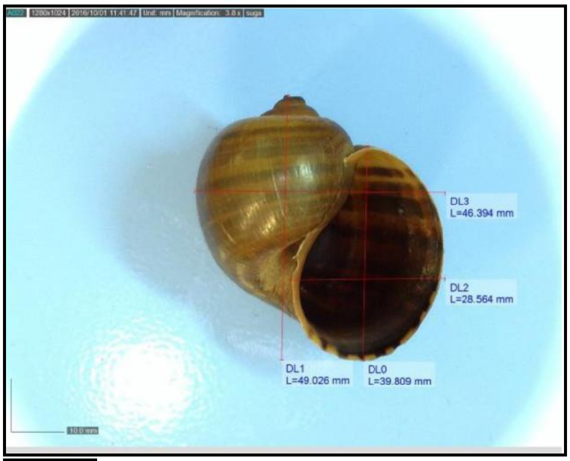

PJ 7

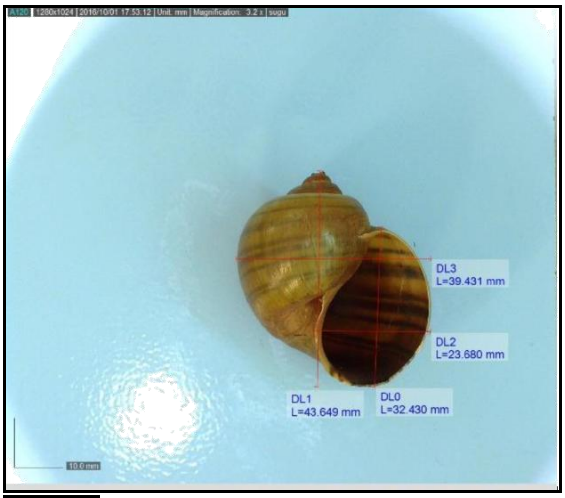

PJ 8

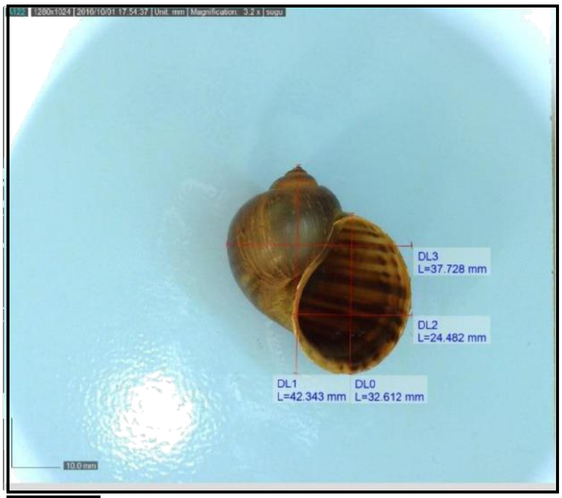

PJ 9

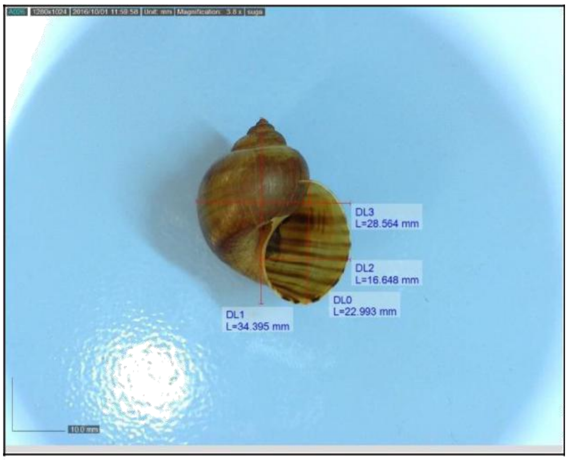

PJ 11

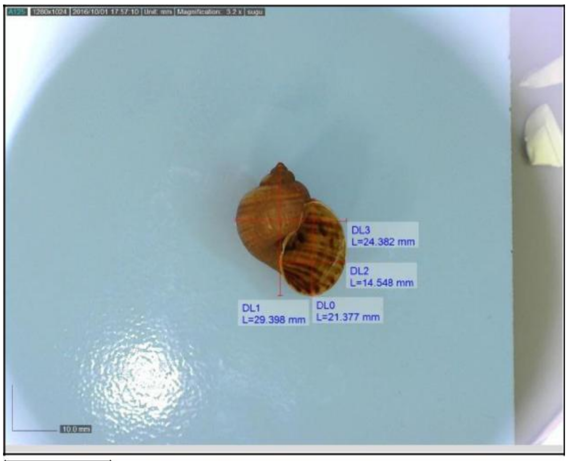

PJ 12

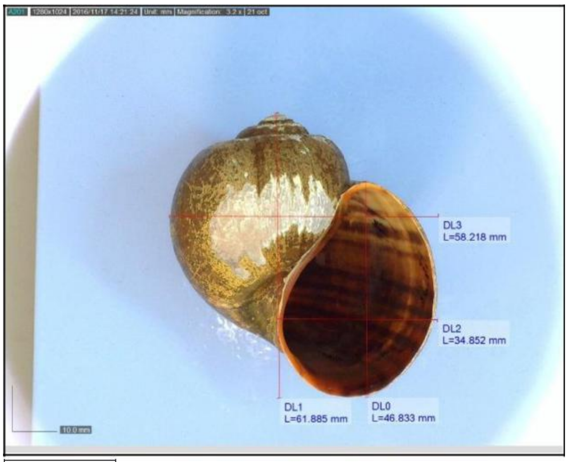

PJ 15

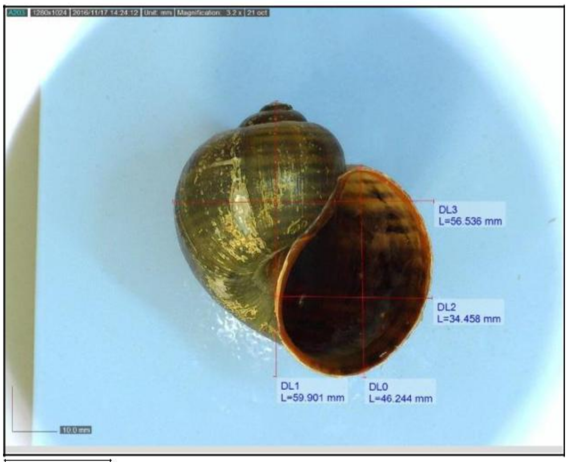

PJ 23

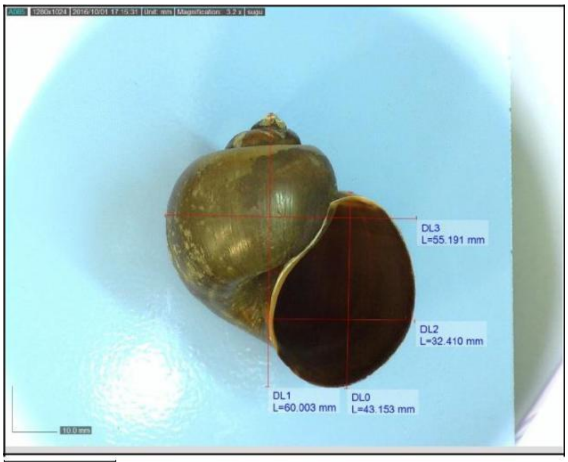

TM 1

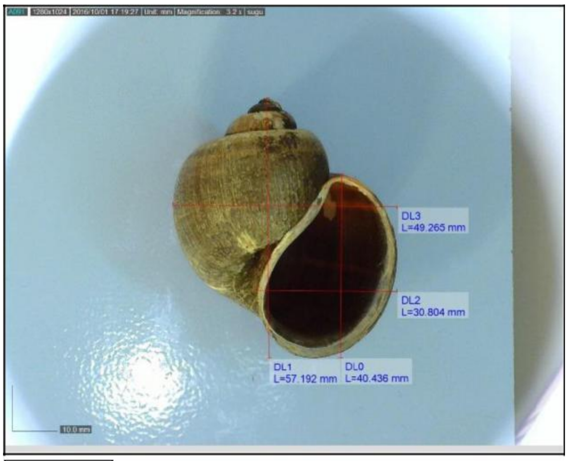

TM 4

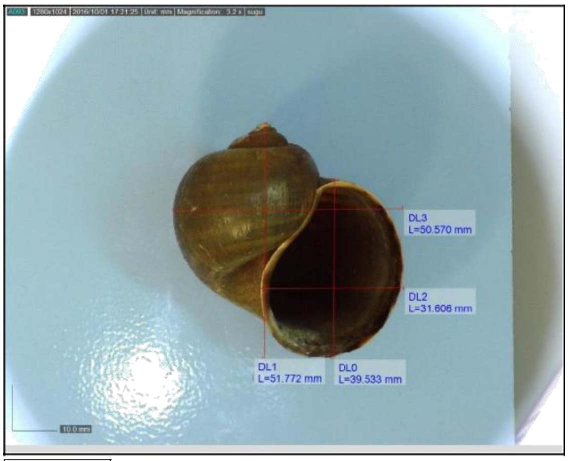

TM 5

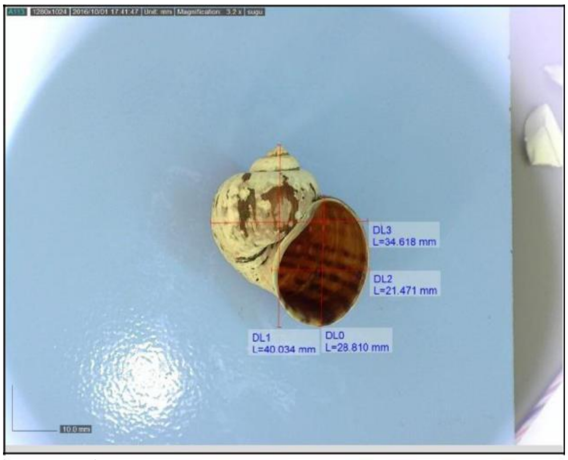

K 3

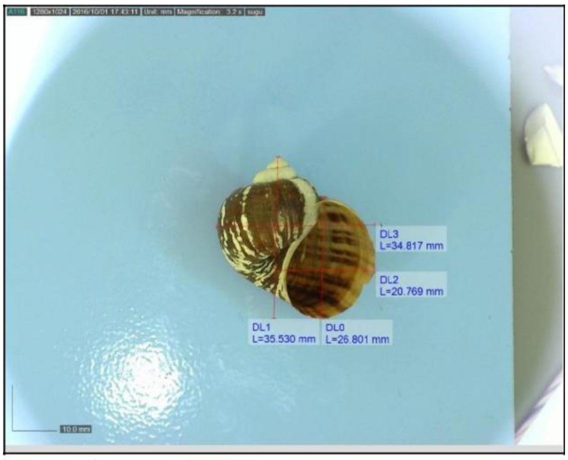

K 4

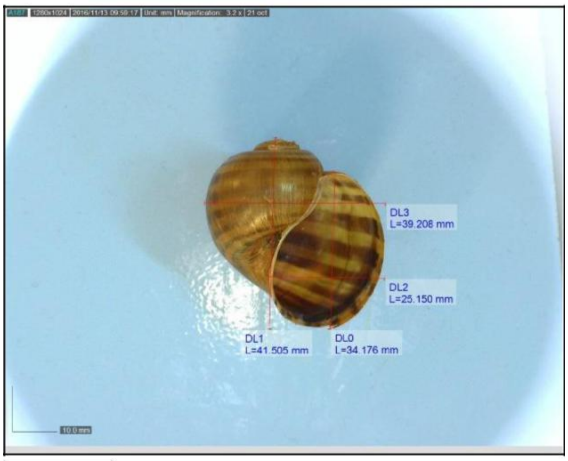

GC 1

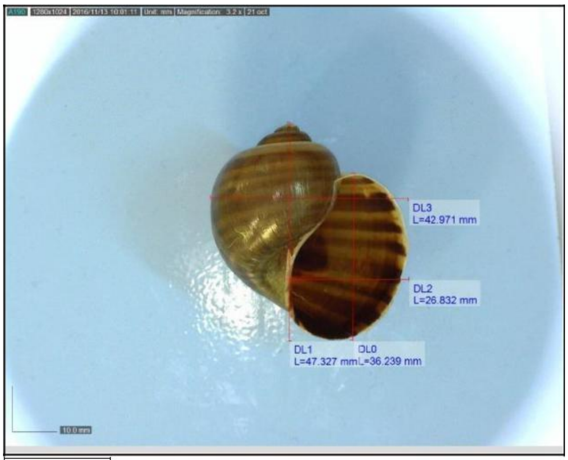

GC 2

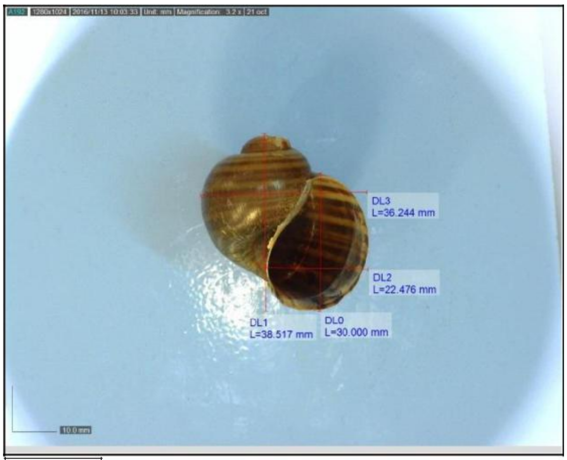

GC 3

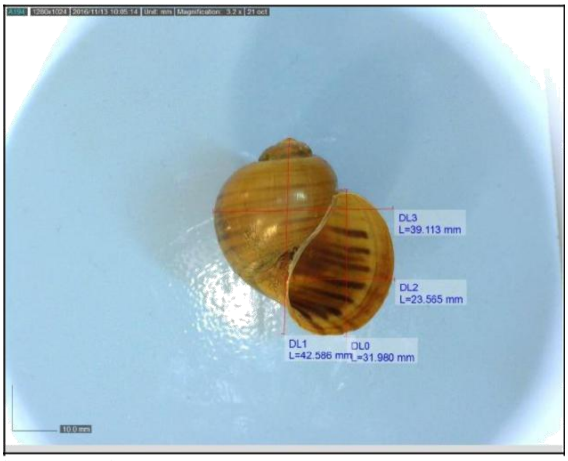

GC 5

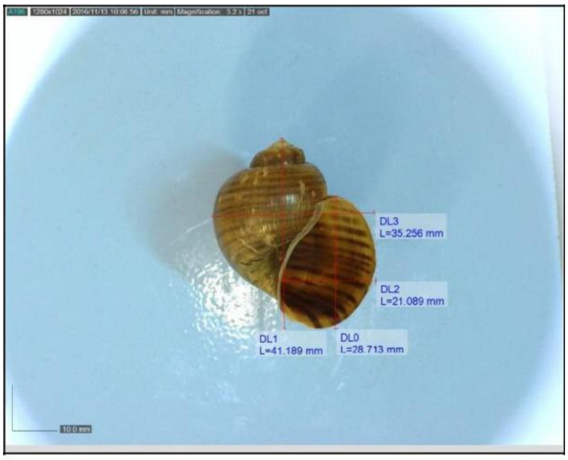

GC 7

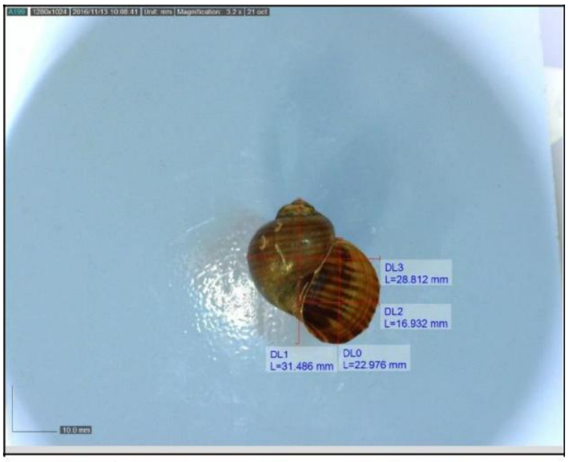

GC 9

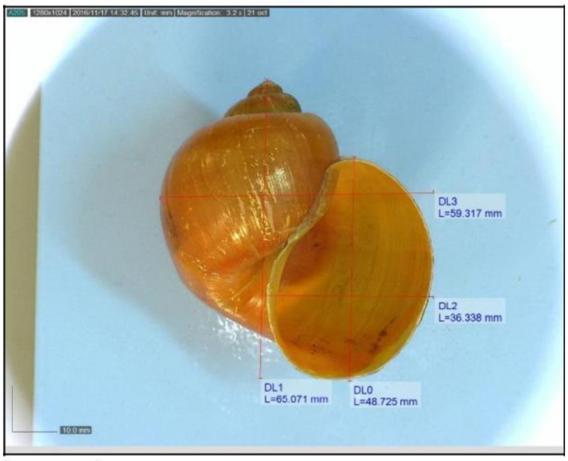

SY 1

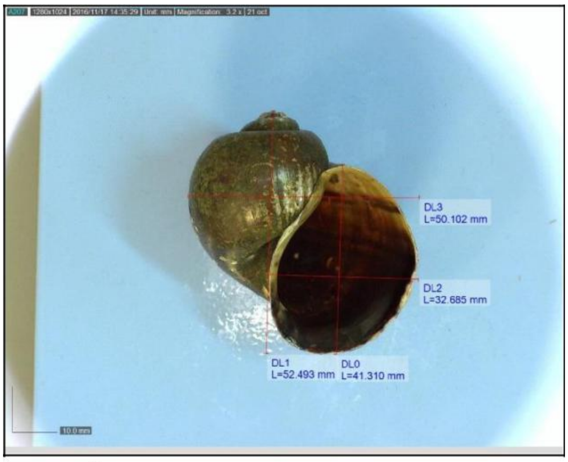

SY 2

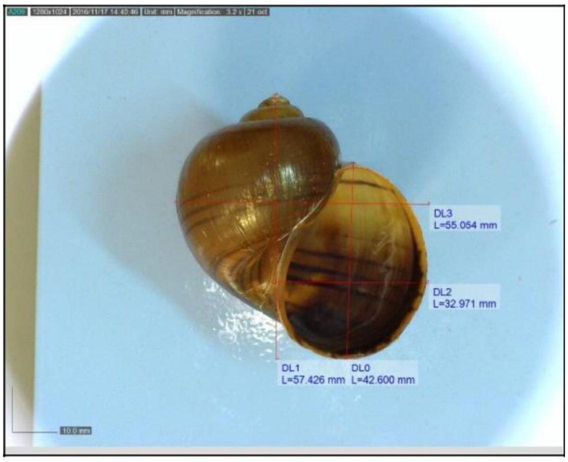

SY 3

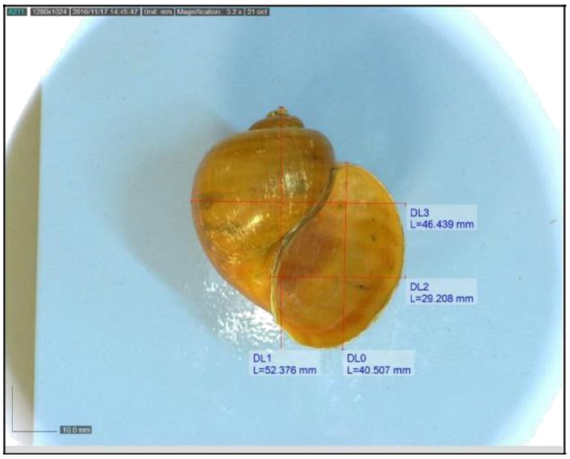
1280x1024
2016/11/17 14:45:47
Unit: mm
Magnification: 3.2 x
21 oct
DL3
L=46.439 mm
DL2
L=29.208 mm
DL1
L=52.376 mm
DL0
L=40.507 mm
10.0 mm

SY 6

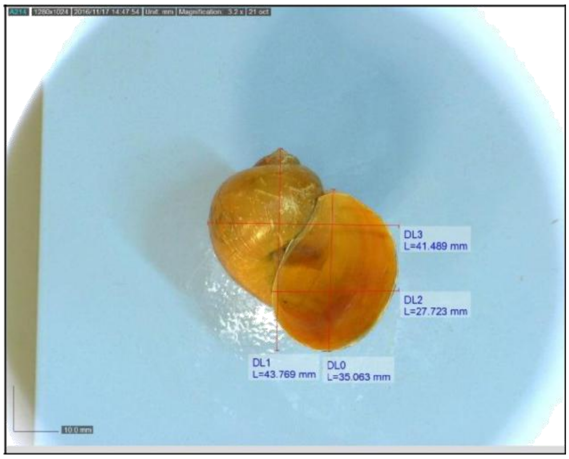
1280x1024
2016/11/17 14:47:54
Unit: mm
Magnification: 3.2 x
21 oct
DL3
L=41.489 mm
DL2
L=27.723 mm
DL1
L=43.769 mm
DL0
L=35.063 mm
10.0 mm

SY 11
