# Supplementary material for: Cryptic diversity: Two morphologically similar species of invasive apple snail in Peninsular Malaysia
Source: PLoS One. 2018 May 7;13(5):e0196582. doi: 10.1371/journal.pone.0196582 (PMC5937749; doi:10.1371/journal.pone.0196582)
Supplement: S3 Fig — (PDF) [file pone.0196582.s005.pdf]

S3 Fig. Apertural views of each *Pomacea canaliculata* specimens.

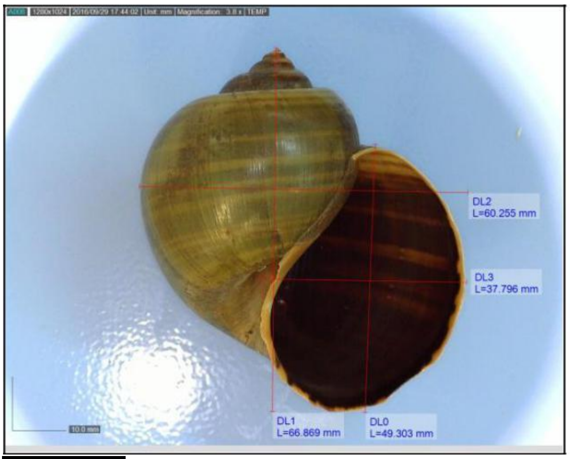

PJ 1

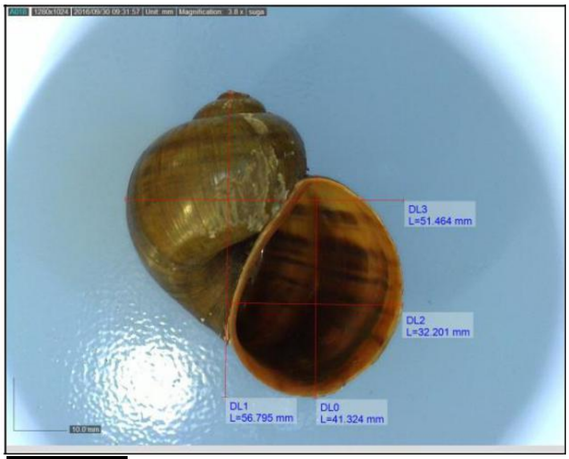

PJ 5

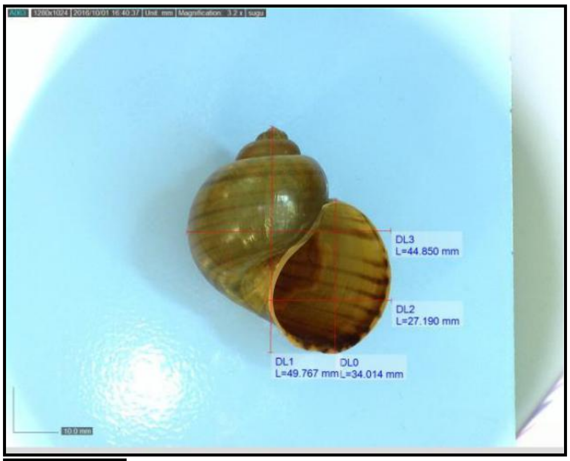

SK 1

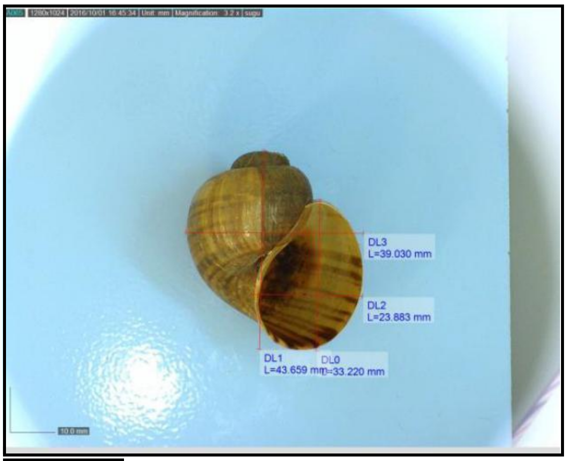

SK 2

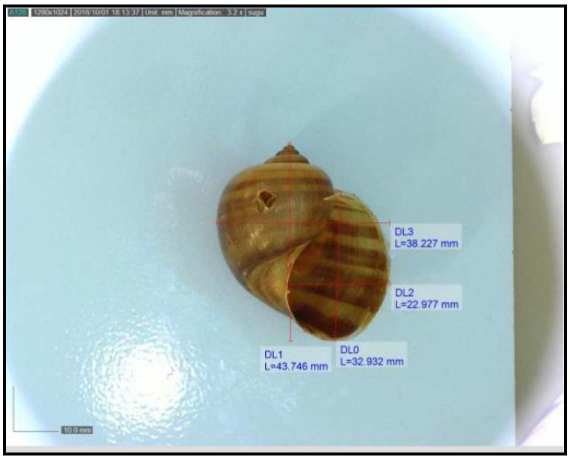

SK 4

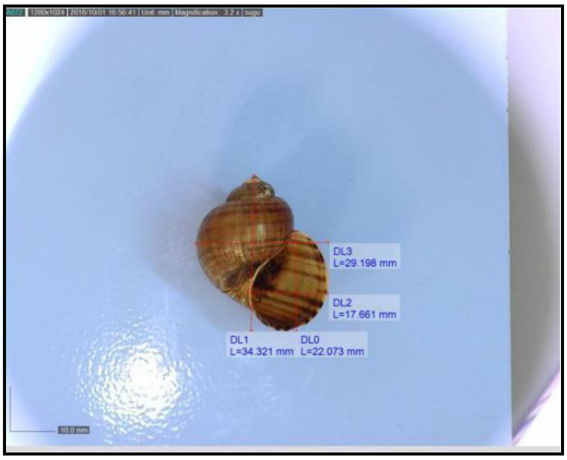

SK 7

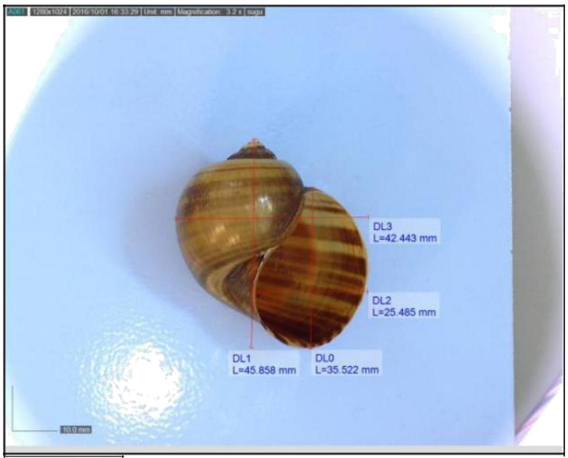

SK 8

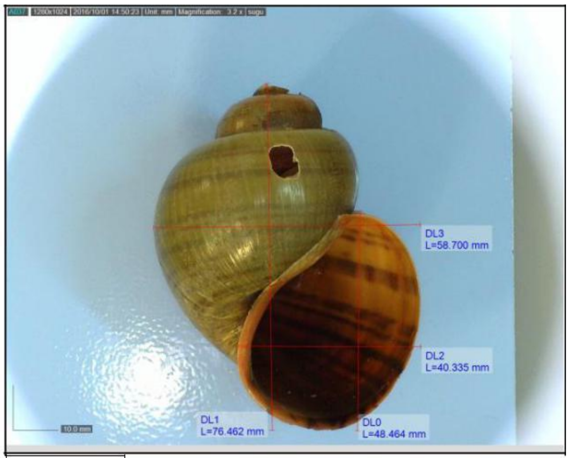

SJ 1

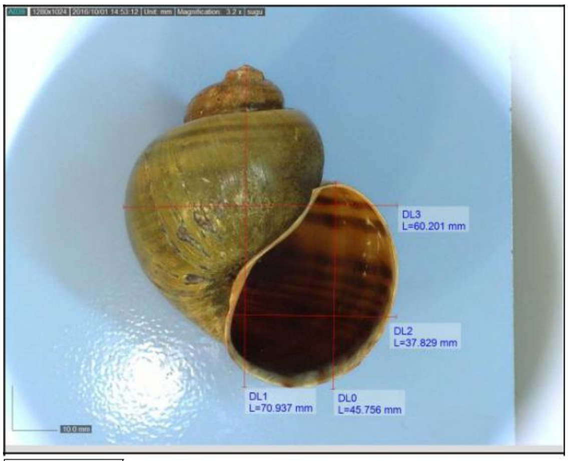

SJ 2

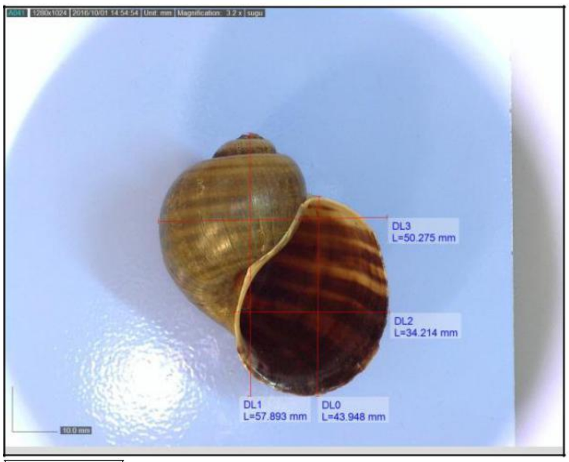

SJ 5

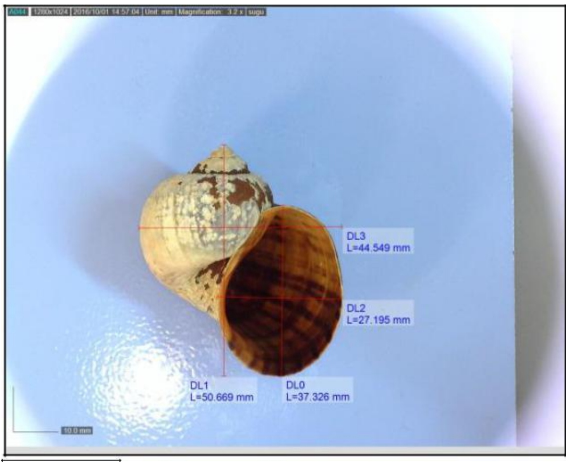

SJ 7

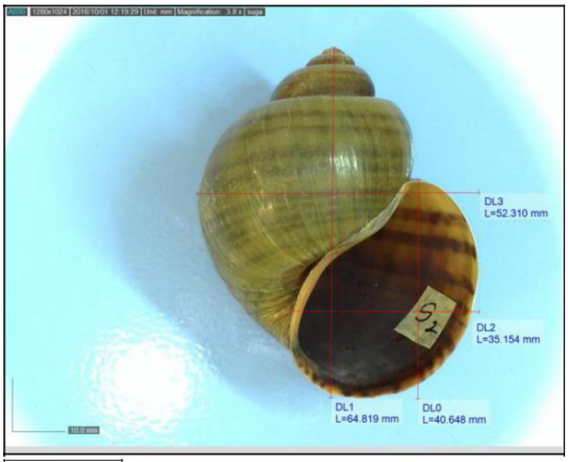

SJ 13

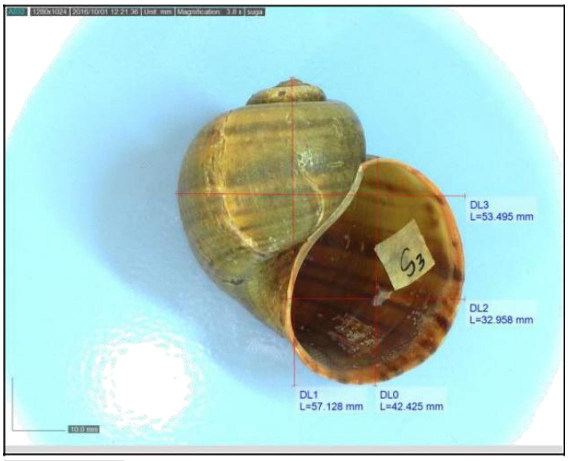

SJ 14

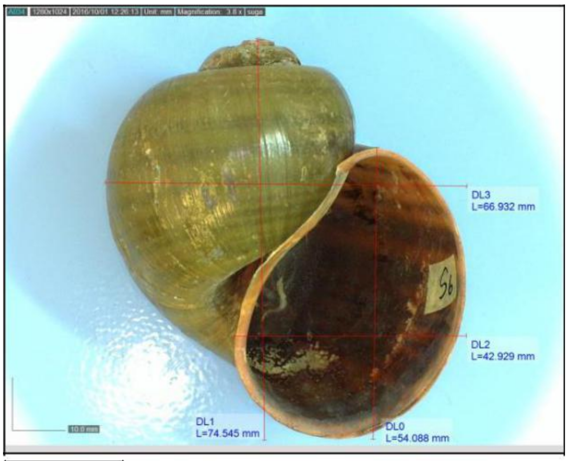

SJ 17

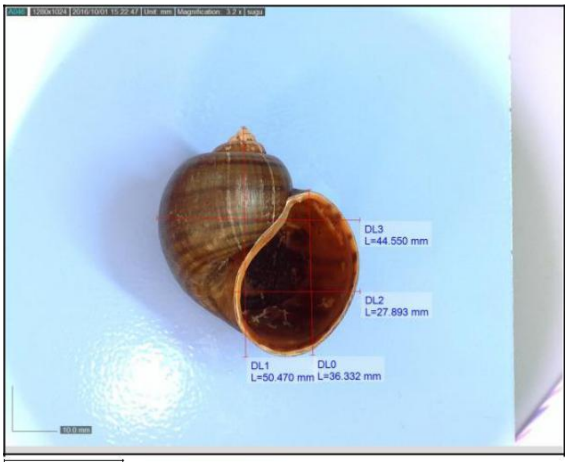

SJ 20

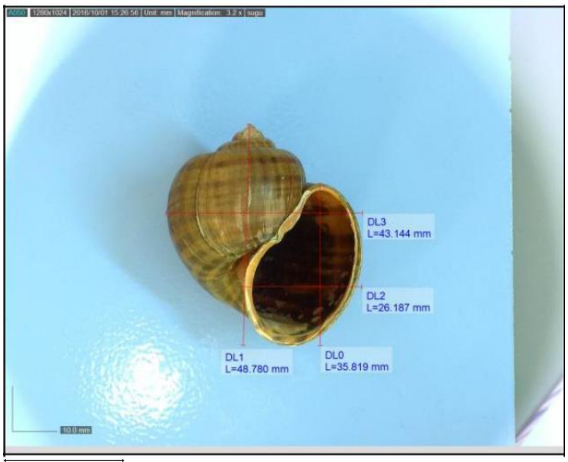

SJ 22

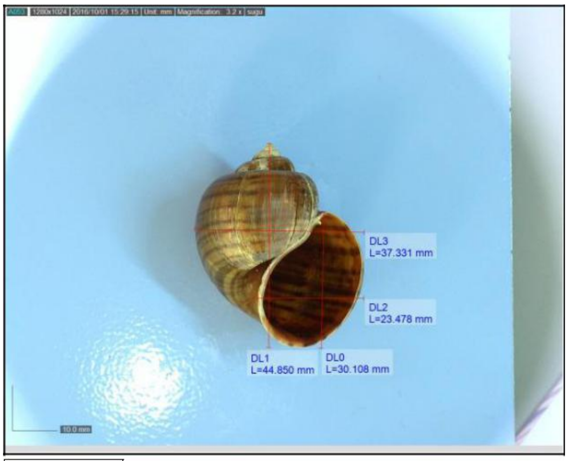

SJ 23

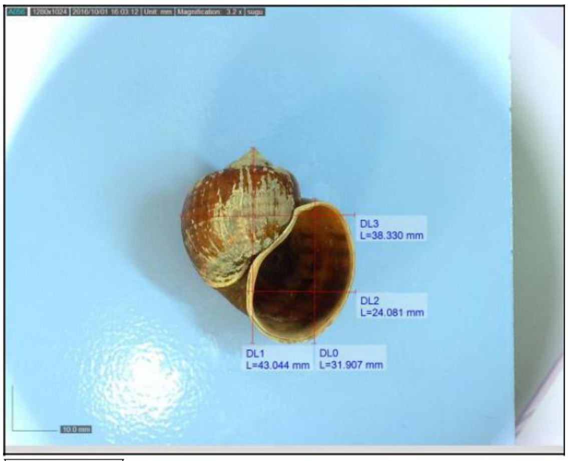

SJ 24

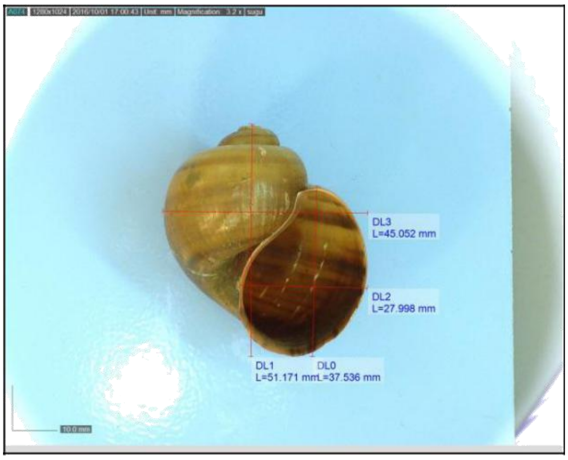

PI 1

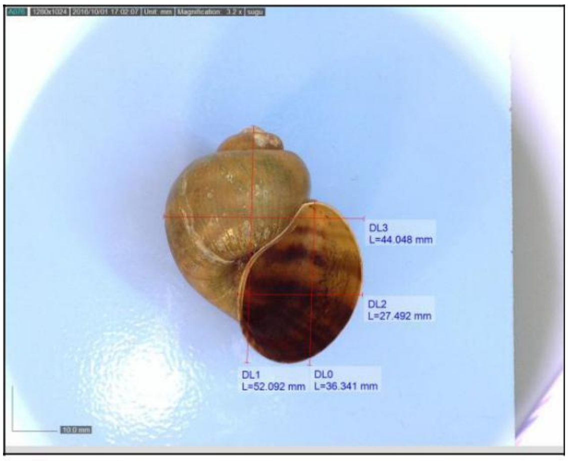

PI 2

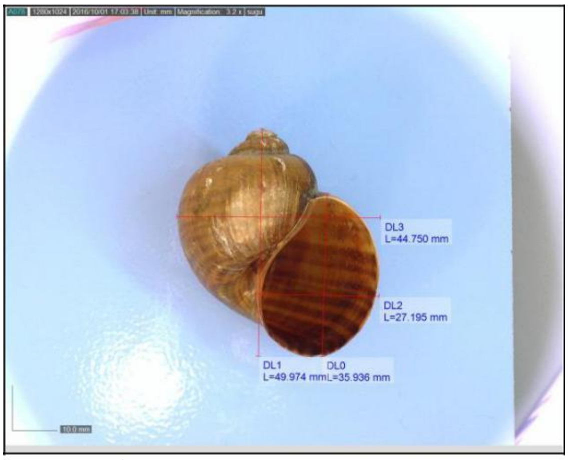

PI 3

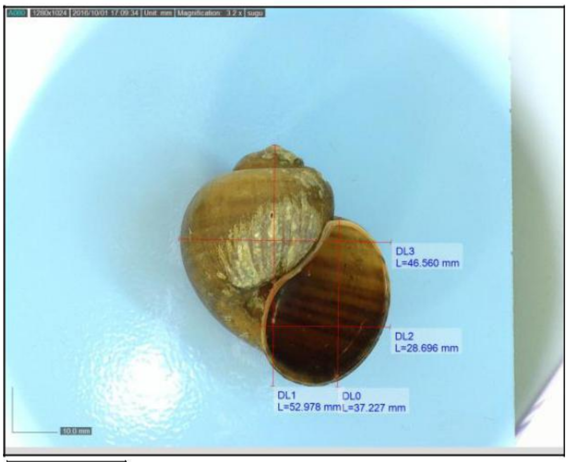

PI 4

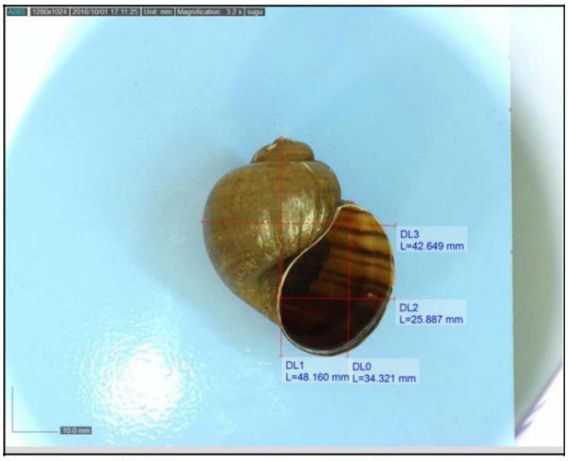

PI 5

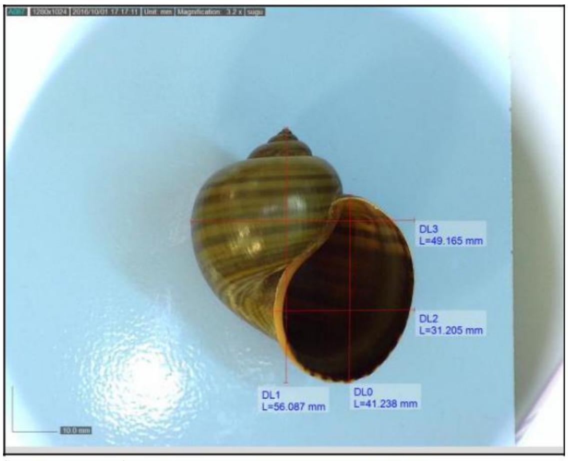

TM 2

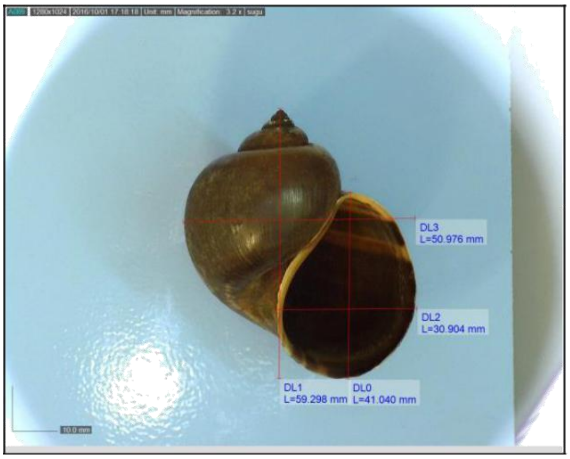
DL3
L=50.976 mm
DL2
L=30.904 mm
DL1
L=59.298 mm
DL0
L=41.040 mm

TM 3

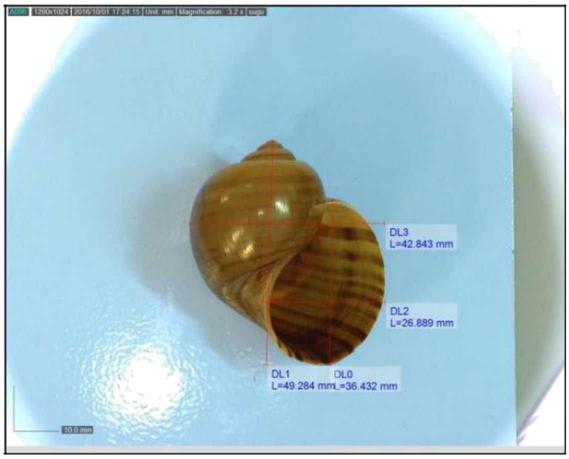
DL3
L=42.843 mm
DL2
L=26.889 mm
DL1
L=49.284 mm
DL0
L=36.432 mm

TM 6
